# Adolescent Ethanol Exposure: Anxiety-Like Behavioral Alterations, Ethanol Intake, and Sensitivity

**DOI:** 10.3389/fnbeh.2020.00045

**Published:** 2020-03-31

**Authors:** Trevor T. Towner, Elena I. Varlinskaya

**Affiliations:** Neurobiology of Adolescent Drinking in Adulthood Consortium (NADIA), Developmental Exposure Alcohol Research Center (DEARC), Department of Psychology, Binghamton University, Binghamton, NY, United States

**Keywords:** adolescence, alcohol, adolescent ethanol exposure, anxiety, ethanol intake, ethanol sensitivity

## Abstract

Adolescence is a developmental period associated with rapid age-specific physiological, neural, and hormonal changes. Behaviorally, human adolescents are characterized by age-typical increases in novelty-seeking and risk-taking, including the frequent initiation of alcohol and drug use. Alcohol use typically begins during early adolescence, and older adolescents often report high levels of alcohol consumption, commonly referred to as high-intensity drinking. Early-onset and heavy drinking during adolescence are associated with an increased risk of developing alcohol use disorders later in life. Yet, long-term behavioral consequences of adolescent alcohol use that might contribute to excessive drinking in adulthood are still not well understood. Recent animal research, however, using different exposure regimens and routes of ethanol administration, has made substantial progress in identifying the consequences of adolescent ethanol exposure that last into adulthood. Alterations associated with adolescent ethanol exposure include increases in anxiety-like behavior, impulsivity, risk-taking, and ethanol intake, although the observed alterations differ as a function of exposure regimens and routes of ethanol administration. Rodent studies have also shown that adolescent ethanol exposure produces alterations in sensitivity to ethanol, with these alterations reminiscent of adolescent-typical ethanol responsiveness. The goal of this mini-review article is to summarize the current state of animal research, focusing on the long-term consequences related to adolescent ethanol exposure, with a special emphasis on the behavioral alterations and changes to ethanol sensitivity that can foster high levels of drinking in adulthood.

## Introduction

Initiation of alcohol use is commonly reported during early (11–15 years of age) adolescence (Faden, 2006; Masten et al., 2009; Morean et al., 2018), and this early initiation is frequently associated with the development of alcohol abuse/dependence later in life (Kuntsche et al., 2016). Several researchers have reported that adolescents who begin drinking at or before the age of 14 are at an elevated risk of becoming alcohol-dependent compared to those who initiate alcohol use at the age of 19 or later (DeWit et al., 2000; Ehlers et al., 2006; Dawson et al., 2008). Likewise, a fast progression from first drink to the first intoxication is a strong predictor and indicator of binge and high-intensity drinking among adolescents (Morean et al., 2014, 2018; Kuntsche et al., 2016; Patrick et al., 2019).

The developing adolescent brain is thought to be particularly vulnerable to alcohol (Olsson et al., 2016; Silveri et al., 2016; Spear, 2018). Thus, early initiation of alcohol use together with high levels of drinking during adolescence (Patrick et al., 2013) can potentially disrupt maturational changes occurring in the brain (Blakemore, 2012; Mills et al., 2014). Therefore, investigations of the consequences of ethanol exposure on the adolescent brain and behavior are critical for understanding the relationship between adolescent drinking and the development of alcohol use disorders later in life.

Rodent models of adolescence allow researchers to determine the consequences of adolescent ethanol exposure that may contribute to the development of alcohol use disorders in adulthood. Behaviorally, consequences of adolescent alcohol exposure include reductions in cognitive flexibility, as well as increases in risk-taking and anxiety, which are associated with multiple neural alterations reviewed in several recent publications (Pascual et al., 2009; Crews et al., 2016, 2019; Spear, 2018). The focus of this mini-review article is on the specific consequences of adolescent alcohol exposure that might foster high levels of ethanol intake later in life and, therefore, become risk factors for the development of alcohol use disorders.

## Adolescent Ethanol Exposure: Impact on Ethanol Intake in Adulthood

Different animal models of ethanol exposure have been used to determine whether adolescent experience with ethanol influences subsequent intake. Voluntary ethanol consumption in laboratory rodents is commonly assessed using a two-bottle choice (2BC) paradigm, in which animals are given free access to water and ethanol, with this paradigm also used for adolescent ethanol exposure. Intake levels in the 2BC paradigm generally do not produce blood ethanol concentrations (BECs) in the binge range (80 mg/dl and higher), but rather in the low-to-moderate range. The original 2BC paradigm has been modified to increase ethanol consumption, *via* models such as the drinking in the dark (DID) paradigm (reviewed in Thiele and Navarro, 2014) and scheduled high-alcohol consumption (SHAC) procedure (Finn et al., 2005). These modified voluntary consumption paradigms implement a restricted time of access to ethanol that allows for elevated ethanol intake resulting in higher BECs, similar to those achieved by forced exposure. Indeed, studies focused on ethanol exposures that produce BECs well into the binge range (100–250 mg/dl) use forced ethanol administration *via* intragastric gavage (IG), intraperitoneal injections (IP), and ethanol vapor inhalation (VI). Findings from studies evaluating the effects of adolescent ethanol exposure on later ethanol intake are presented in Table 1.

**Table 1:** Effects of adolescent ethanol exposure on ethanol intake.

		Exposure					
Strain	Sex	Route and Dose	Pattern	Timing and Duration (days)	Test	Results	Reference
Sprague–Dawley rats	Male	IP 3.0 g/kg	Intermittent	P30–43 (14) P45–58 (14)	2BC Operant SA	**Increased intake** after P30–43 exposure	Alaux-Cantin et al. (2013)
	Male	IP 4.0 g/kg	Intermittent	P24–33 (10) P69–78 (10)	2BC	No change in intake	Broadwater et al. (2011)
	Male Female	1 bottle with 10% EtOH in SS	Intermittent	P28–42 (15)	30-min access to 10% sweet EtOH	**Increased intake** of a familiar solution during initial sessions	Broadwater et al. (2013)
	Male	Single bottle with fade on to 20% EtOH	Continuous	P35–250 (215) P75–290 (215)	Continuous single bottle access	**Increased intake** for adolescent-onset animals	Fernandez et al. (2016)
	Male Female	IG 1.5, 3.0, or 5.0 g/kg	Intermittent	P28–45 (18)	2BC	**Increased intake**	Maldonado-Devincci et al. (2010)
	Male	IP 2.0 g/kg	Intermittent	P28–41 (14)	2BC	**Increased intake**	Pandey et al. (2015)
	Male	IP 2.0 g/kg	Intermittent	P28–41 (14)	2BC	**Increased intake**	Sakharkar et al. (2019)
	Male	VI	Consecutive	P30–39 (10)	Free choice operant	No change in intake	Slawecki and Betancourt (2002)
	Male Female	IG 3.5 g/kg	Intermittent	P25–45 (21)	Social drinking	No change in intake	Varlinskaya et al. (2017)
	Male	2BC 10% EtOH	Continuous	P27–90 (64) P70–90 (20)	2BC	No change in intake	Vetter et al. (2007)
	not stated	2BC 5% EtOH sweetened	Variable	P22–50 (29)	Operant SA	No change in intake	Williams et al. (2018)
Wistar rats	Male	2BC 20% EtOH	Intermittent	P26–59 (34) P92–125 (34)	Operant SA	Increased SA regardless of exposure age	Amodeo et al. (2017)
	Male Female	2BC 20% EtOH + VI	Variable	P22–67 (46)	2BC	**Increased intake**	Amodeo et al. (2018)
	Male	1 bottle 5% EtOH	Intermittent	P40–90 (51)	2BC	**Increased intake**	Blomeyer et al. (2013)
	Male	2BC 10% EtOH + VI	Variable	P29–100 (72)	2BC	**Increased intake**	Criado and Ehlers (2013)
	Female	3BC 5 and 20% EtOH	Continuous	P31–87 (56) P71–127 (56)	3BC	Adult onset consumed more ethanol than adolescent onset	Fullgrabe et al. (2007)
	Male Female	4BC 5, 10, and 20% EtOH	Continuous	P19–28 (10) P28–37 (10) P90–99 (10)	4BC	No difference in intake	García-Burgos et al. (2009)
	Male	1 bottle of 5% sweetened EtOH Operant SA IP 2 g/kg	Variable	P27–39 (13) P28–42 (14)	Single bottle 30-min access or operant SA	No difference in adult intake following voluntary ethanol consumption; decrease in adult intake following adolescent ethanol exposure *via* injection	Gilpin et al. (2012)
	Male	1 bottle 8% EtOH	Consecutive during the dark phase (12 h/day)	P51–58 (7)	2BC	**Increased intake**	Milivojevic and Covault (2013)
	Male	IP 3.0 g/kg	Intermittent	P25–38 (14)	2BC (24 h and limited access)	**Increased intake**	Pascual et al. (2009)
	Female	2BC 15% EtOH	Continuous	P30–60 (30)	Operant SA	**Increased acquisition**	Rodd-Henricks et al. (2002)
	Male	3BC 5 and 20% EtOH	Continuous	P31–71 (40)	3BC	Adult-onset consumed more ethanol than adolescent-onset	Siegmund et al. (2005)
	Female	2BC 15% EtOH	Continuous	P30–60 (30)	Operant SA	**Increased acquisition**	Toalston et al. (2015)
	Male	Traverse runway to have free access to varying % + yoked controls	Consecutive	P29–54 (25)	2BC	Adolescents that navigated runway to earn ethanol reward had **increased intake** in adulthood compared to yoked controls	Walker and Ehlers (2009)
Long-Evans rats	Male	VI	Intermittent	P28–42 (14)	Operant SA	**Increased intake**	Gass et al. (2014)
	Male	2BC 20% EtOH	Intermittent	P23–56 (24)	2BC	No changes in intake	Moaddab et al. (2017)
	Male	VI	Intermittent	P28–44 (16)	2BC Operant SA	No changes in intake	Nentwig et al. (2019)
	Male Female	1 bottle 5% or 10% EtOH	Continuous	P21–70 (50)	2BC	**Male mice increased intake**; Female mice decreased intake	Siciliano and Smith (2001)
C57 mice	Male	2BC 15% EtOH+ VI	Variable	P30–57 (28) P70–97 (28)	2BC	**Increased intake** but no effect of age of exposure	Carrara-Nascimento et al. (2013)
	Male Female	SHAC 5% EtOH	Limited intermittent	P28–49 (21) P56–77 (21)	2BC	No changes in intake	Cozzoli et al. (2014)
	Male	2BC 10% EtOH	Continuous	P28–49 (21) P42–63 (21)	2BC	No changes in intake	Hefner and Holmes (2007)
	Male	2BC 10% EtOH	Continuous	P24–112 (88) P56–112 (56)	2BC	**Increased intake** with adolescent onset of ethanol consumption	Ho et al. (1989)
	Female	VI	Consecutive	P28–56 (28) P56–84 (28)	2BC	No changes in intake	Jury et al. (2017)
	Male	2BC 15% EtOH+ VI	Variable	P28–70 (42) P56–98 (42)	2BC	No change in intake for adolescent exposed; adult CIE exposed increased intake	Jury et al. (2017)
	Male	4BC-DID 5, 10, 20, and 40% EtOH	Consecutive limited 2-h access	P28–41 (14) P56–69 (14)	DID	**Increased intake** following adolescent ethanol exposure	Lee et al. (2017)
	Male Female	DID 20% EtOH	Consecutive limited 2 h access	P28–42 (14)	DID	**Increased intake**	Moore et al. (2010)
	Male Female	SHAC 5% EtOH	Limited intermittent	P26–47 (21) P58–79 (21)	DID 2BC	No difference in intake during DID; **increased intake** for both sexes during the 2BC following adolescent exposure	Strong et al. (2010)
BALB	Male Female	2BC 10% EtOH; 1 bottle 10% EtOH; Gradual 0.5–10% EtOH	Continuous	P35–84 (49)	2BC	BALB/cByJ **increased intake** regardless of method BALB/cJ **increased intake** following gradual concentration change	Blizard et al. (2004)
	Male Female	IG 2.2 g/kg	Consecutive	P22–25 (4)	DID	**Increased intake**	Jacobsen et al. (2018)
HS/Ibg (HAP2 or WSC1)	Male Female	2BC 10% EtOH	Continuous	P28–42 (14) P60–74 (14)	2BC	**Increased intake** over initial test days but no effect of age of exposure	O’Tousa et al. (2013)
	Male Female	2BC 6% EtOH	Continuous	P28–84 (56) P70–126 (56)	2BC	**Increased intake** but no effect of age of exposure	Tambour et al. (2008)

EtOH, Ethanol; 2BC, Two bottle choice; 3BC, Three bottle choice; 4BC, Four bottle choice; IP, Intraperitoneal; IG, Intragastric Gavage; VI, Vapor Inhalation; SS, Super sac; DID, Drinking in the Dark; SHAC, Scheduled High Alcohol Consumption; SA, Self-Administration. *Testing took place in adolescence for the adolescent exposed group. The bold text in the table represents results in which increases in ethanol consumption were found.

More than half of the studies outlined in Table 1 demonstrate that adolescent ethanol exposure results in persistent increases in ethanol intake in adulthood, findings across multiple models of exposure and intake assessment methods. For example, although Blomeyer et al. (2013) exposed male, Wistar rats *via* a 2BC paradigm during mid-adolescence, and Pandey et al. (2015) administered ethanol IP (2 g/kg) in male, Sprague–Dawley rats, both studies demonstrated higher intakes in ethanol-exposed animals relative to their age-matched controls. Similarly, Gass et al. (2014) reported increased ethanol intake in adulthood following adolescent VI exposure. Some studies have also shown increased intake in adulthood following a combination of voluntary (2BC) and forced (VI) ethanol exposures (Criado and Ehlers, 2013; Amodeo et al., 2018). However, 19 of the studies reviewed found either no effects of adolescent ethanol exposure, more pronounced effects of adult exposure on ethanol intake, or an equivalent response following adolescent and adult exposures (see Table 1).

Several factors potentially contribute to these inconsistent findings, with exposure duration and exposure timing representing two important variables that vary drastically between the different studies. Throughout the reviewed literature, exposure duration ranged from 4 days (Jacobsen et al., 2018) to as long as 8 weeks (Fullgrabe et al., 2007) during adolescence and in some cases well into adulthood (Fernandez et al., 2016). The results of the only study that directly addressed the issue of exposure timing within the adolescent period on ethanol intake later in life demonstrated that early adolescent males (postnatal days 30–43) are more vulnerable to ethanol-exposure-related increases in ethanol intake than their more mature (postnatal days 45–58) adolescent counterparts (Alaux-Cantin et al., 2013).

Only a third of the studies (5 out of 16) that used voluntary ethanol consumption, such as 2BC or similar models of adolescent exposure, demonstrated increased ethanol intake later in life. Very few studies reported BEC data during the voluntary consumption exposure phase, and those that did generally observed low BECs that ranged from 0 to 100 mg/dl with averages around 25–35 mg/dl (Gilpin et al., 2012; Broadwater et al., 2013; Amodeo et al., 2017; however see O’Tousa et al., 2013). However, findings of studies implementing voluntary consumption in which higher BECs (80–200 mg/dl) were achieved, commonly from DID and SHAC procedures, generally supported elevated intake levels in adulthood. Of the studies that report increased ethanol intake in adulthood, forced adolescent exposure appears to more effectively increase ethanol intake than voluntary exposure models. Indeed, more than half (11 out of 19) of the studies that employed forced exposure paradigms reported exposure-related increases in ethanol intake. Together, these findings suggest that to enhance ethanol intake later in life, adolescent exposure models, both voluntary and forced, should produce BECs well into the binge range.

Many studies that reported increases in ethanol intake following adolescent ethanol exposure did not include other age groups for comparison. Of the studies that included adolescents and adults, previous exposure to ethanol tended to increased ethanol intake later in life regardless of exposure timing (Hefner and Holmes, 2007; Tambour et al., 2008; Strong et al., 2010; Carrara-Nascimento et al., 2013; O’Tousa et al., 2013; Amodeo et al., 2017) or following adult exposure only (Fullgrabe et al., 2007; Jury et al., 2017), suggesting that exposure-related increases in ethanol consumption may not be specific to adolescent exposure. Therefore, it is still not clear whether adolescents are more vulnerable than adults to ethanol-exposure-related increases in ethanol intake.

The studies conducted to date indicate that many factors contribute to the effects of adolescent ethanol exposure on ethanol intake later in life, including exposure regiments (continuous vs. intermittent), exposure duration, exposure mode (voluntary vs. forced), exposure levels, BECs achieved, and strain. Given their respective effects and contributions, all these factors should be considered in future studies.

## Ethanol Sensitivity Following Adolescent Ethanol Exposure

In general, adolescent laboratory rodents are less responsive than their adult counterparts to adverse effects of ethanol that may curb ethanol intake. These adverse effects of ethanol include social inhibition (Varlinskaya and Spear, 2002), sedation (Moy et al., 1998; Silveri and Spear, 1998; Draski et al., 2001), motor impairment (White et al., 2002; Ramirez and Spear, 2010), and aversion (Vetter-O’Hagen et al., 2009; Anderson et al., 2010; Schramm-Sapyta et al., 2014; Saalfield and Spear, 2015, 2019). In contrast, adolescent rats are uniquely responsive to social facilitation induced by low doses of ethanol (Varlinskaya and Spear, 2002, 2007, 2015; Trezza et al., 2009; Willey et al., 2009), with some evidence also suggesting higher responsiveness to the rewarding effects of ethanol during adolescence than in adulthood (Pautassi et al., 2008). Adolescent ethanol exposure produces alterations in responsiveness to ethanol that resemble these adolescent-typical ethanol sensitivities. This retention of adolescent-typical responding to ethanol has been termed as the “lock-in” effect of adolescent ethanol exposure (reviewed in Spear and Swartzwelder, 2014). The “locking in” of adolescent-typical responding to ethanol effects may play a substantial role in increased ethanol intake in adulthood following adolescent exposure.

Effects of chronic adolescent exposure to ethanol on ethanol-induced sedation indexed *via* the loss of the righting reflex (LORR) have been assessed in laboratory rodents. For instance, adult male rats exposed to ethanol during adolescence (P30–48, 1, 2, 3 or 4 g/kg ethanol, IP) and challenged with a hypnotic ethanol dose regained their righting reflex more rapidly than did their non-exposed counterparts (Matthews et al., 2008), with these alterations evident only following high exposure doses of ethanol (3 and 4 g/kg). These results were also replicated by the same group (Matthews et al., 2017) and others using mice (Jury et al., 2017). However, similar decreases in LORR duration were evident following adult exposure as well (Jury et al., 2017). These findings suggest that adolescent ethanol exposure results in relative insensitivity to ethanol-induced sedation, although the development of metabolic tolerance to ethanol cannot be ruled out. Indeed, the development of metabolic tolerance has been reported following adolescent ethanol exposure (Silvers et al., 2003). When adolescent and adult male rats were repeatedly exposed to an ethanol dose of 4 g/kg and challenged with the same dose 24 h after the last exposure, adult rats, but not their adolescent counterparts, demonstrated chronic tolerance to the sedative effects of ethanol that appeared to be metabolic, but not functional (Broadwater et al., 2011). Evidence of decreased sensitivity to ethanol-induced sedation associated with adolescent ethanol exposure came from the study of Quoilin et al. (2012): female Swiss mice exposed to ethanol during adolescence regained the righting reflex at higher BECs than controls.

Adult animals exposed to ethanol during adolescence become relatively insensitive to the aversive effects of ethanol assessed *via* ethanol-induced conditioned taste aversion (CTA). Diaz-Granados and Graham (2007) exposed adolescent male mice to ethanol vapor either continuously or intermittently and found attenuated CTA to ethanol later in life, with intermittent exposure producing greater attenuation and adult exposure not producing similar effects. Saalfield and Spear (2015), assessing the impact of ethanol exposure (4.0 g/kg, IG) during early (P25–45) and late (P45–65) adolescence on ethanol-induced CTA in male rats, found that both adolescent exposures resulted in decreased sensitivity to the aversive effects of ethanol. Alaux-Cantin et al. (2013) also found that male rats exposed to ethanol during early adolescence (3 g/kg, IP, P30–43) demonstrated attenuated ethanol-induced CTA in adulthood. The reductions in sensitivity to ethanol CTA following adolescent ethanol exposure appear to be sex-specific, with only male Long-Evans rats, but not females, demonstrating an attenuated CTA in adulthood following adolescent exposure (Sherrill et al., 2011).

Adolescent ethanol exposure (P25–45, IG, 4 g/kg) of Sprague–Dawley male rats resulted in precipitation of adolescent-typical responding to acute ethanol challenge with social facilitation (i.e., ethanol-induced increases in peer-directed social behavior) when these males were tested in adulthood (Varlinskaya et al., 2014). Enhanced sensitivity to ethanol reinforcement indexed *via* a significant leftward shift in the dose-response curve for ethanol self-administration into the posterior ventral tegmental area following adolescent ethanol exposure (4 g/kg IG, P28–48) was also evident in adult male and female Wistar rats, as well as in alcohol-preferring (P) male rats (Hauser et al., 2019). Carrara-Nascimento et al. (2014) showed that adult male Swiss mice exposed to ethanol during adolescence displayed a robust CPP to 2.0 g/kg ethanol, whereas adult exposure decreased sensitivity to the reinforcing properties of ethanol. Similarly, BALB/c adult mice demonstrated enhanced sensitivity to ethanol-induced CPP following only four exposures to ethanol given during the juvenile period on P22–25 (Jacobsen et al., 2018).

Taken together, the experimental findings demonstrate that exposure to ethanol during adolescence changes sensitivity to many ethanol effects later in life, decreasing sensitivity to adverse effects of ethanol and making adult laboratory rodents more sensitive to stimulatory and rewarding properties of ethanol. This pattern of sensitivity to the adverse and desired ethanol effects, reminiscent of that typically shown by adolescent rodents, may allow adult animals to ingest higher amounts of ethanol without experiencing negative consequences.

## Adolescent Alcohol Exposure: Anxiety-Like Behavioral Alterations

Adolescents and young adults who engage in problematic drinking often drink for enhancement of positive emotional states or alleviation of negative affective states (Ham and Hope, 2003; Kuntsche et al., 2006). The association between negative reinforcement and alcohol use has been shown to become stronger in individuals with alcohol use disorder, with no changes evident in the association between positive reinforcement and alcohol consumption (Cho et al., 2019). Indeed, available research suggests relatively strong associations between adolescent alcohol use and increased prevalence of anxiety and depression disorders in adulthood (Rohde et al., 2001; Jeanblanc, 2015). In older adults, alcohol use disorder is frequently comorbid with depression and anxiety (Vorspan et al., 2015; Wiener et al., 2018). Therefore, the assessment of affective behavioral alterations in animal models of adolescent alcohol exposure seems utterly important.

Increases in anxiety-like behavior have been reported using different models of adolescent ethanol exposure. For instance, adult male Sprague–Dawley rats exposed to 2 g/kg ethanol (Kokare et al., 2017; Kyzar et al., 2017, 2019; Sakharkar et al., 2019) or 4 g/kg ethanol (Van Skike et al., 2015) given IP during adolescence, as well as male Long-Evans rats exposed IG to a 1.5 g/kg ethanol dose (Loxton and Canales, 2017), demonstrated elevated levels of anxiety-like behavior when tested on the elevated plus-maze (EPM). Our recent findings indicated that IG ethanol exposure of Sprague–Dawley males and females during early/mid-adolescence (P25–45) results in enhanced anxiety-like behavior on the EPM in adulthood regardless of sex (Varlinskaya et al., 2019). However, only males demonstrated enhanced anxiety-like behavior on the EPM following late-adolescent/emerging-adulthood exposure (P45–65), suggesting that the effects of ethanol exposure are sex- and exposure-timing dependent.

However, Torcaso et al. (2017), exposing male Wistar rats IG to ethanol (3 g/kg, P37–44) reported no behavioral changes on the EPM, whereas other researchers have demonstrated that adolescent ethanol exposure resulted in decreases of anxiety-like behavior on the EPM (Gilpin et al., 2012; Gass et al., 2014). For example, Long-Evans male rats were intermittently exposed to ethanol *via* VI during early-mid adolescence (P28–P42), and this exposure regimen resulted in decreased anxiety, as indexed by increased open arm behavior evident in adult rats (Gass et al., 2014). Similarly, adolescent (P28–P42) ethanol exposure of male Wistar rats *via* self-administration increased percent open arm time on the EPM when these males were tested in adulthood (Gilpin et al., 2012). These inconsistent results are likely associated with procedural differences such as rat strain, route of ethanol administration, phase of light/dark cycle during testing, and pre-test manipulations (see Hogg, 1996; Carobrez and Bertoglio, 2005). For example, the two studies that reported decreased anxiety-like behavior on the EPM tested animals in low-light conditions during the dark part of the light/dark cycle, conditions that may reduce anxiety-like behavior (Gilpin et al., 2012; Gass et al., 2014). It is possible that the observed increases in open arm entries and/or open arm time reflect disinhibition but not decreases in anxiety-like behavior, since the characteristics of the test situation determine whether anxiety or disinhibition are manifested in the EPM (Ennaceur, 2014).

Anxiety-like alterations associated with adolescent ethanol exposure were reported for other tests of anxiety as well. Experiments using the light/dark box have shown that adolescent ethanol exposure increases time spent in the dark portion of the apparatus and decreases entries into the light side in male Sprague–Dawley rats (Pandey et al., 2015; Vetreno et al., 2016; Sakharkar et al., 2019). Anxiety-like behavior in the open-field (Coleman et al., 2014; Yan et al., 2015) and marble-burying test (Lee et al., 2017) have also been enhanced following adolescent ethanol exposure of Sprague–Dawley males (Yan et al., 2015) and male C57BL/6J mice (Coleman et al., 2014; Lee et al., 2017). When tested in adulthood, male Sprague–Dawley rats exposed to ethanol during early-mid adolescence demonstrated social anxiety-like behavioral alterations indexed *via* decreases in social investigation and social preference (Varlinskaya et al., 2014), with no changes in social behavior evident following late adolescent ethanol exposure.

Increased anxiety-like behavior following adolescent ethanol exposure may also contribute to increases in ethanol intake due to the anxiolytic properties of ethanol. Although links between anxiety and alcohol consumption have been commonly reported in humans (Vorspan et al., 2015) and laboratory rodents (Pelloux et al., 2015), it remains to be investigated whether animals that demonstrate increases in anxiety following adolescent ethanol exposure drink more ethanol for its negatively reinforcing, anxiolytic effects.

## Conclusions

Although adolescent alcohol exposure is associated with behavioral alterations and changes in ethanol sensitivity, it is still not clear whether these alterations contribute to increases in ethanol intake. Considering that very few studies assessing changes in ethanol intake following adolescent ethanol exposure included both sexes, the question of whether responding to ethanol exposure during adolescence differs in males and females remains unanswered. Furthermore, among the studies that assessed changes in ethanol intake, only a limited number included both adolescent and adult ethanol exposure conditions (see Table 1), producing mixed results and not allowing to conclude that enhanced ethanol intake in adulthood is specific to adolescent ethanol exposure. The impact of ethanol exposure timing within the adolescent period (i.e., during early vs. late adolescence) on ethanol intake and sensitivity is still not well understood, and this important issue should also be addressed in future studies (Spear, 2015). The relative insensitivity of adolescents to the acute effects of ethanol is related in part to age differences in compensatory responses, including acute tolerance, that serve to counteract ethanol-induced impairment. Therefore, it is important to investigate whether adolescent ethanol exposure decreases sensitivity to the adverse effects of acute ethanol by enhancing the development of acute tolerance. More studies are needed for a better understanding of the consequences of alcohol exposure during adolescence that might contribute to heavy drinking later in life and put individuals at risk for the development of alcohol use disorders.

## Author Contributions

TT and EV contributed equally.

## Conflict of Interest

The authors declare that the research was conducted in the absence of any commercial or financial relationships that could be construed as a potential conflict of interest.
